# Vitamin D signaling inhibits HBV activity by directly targeting the HBV core promoter

**DOI:** 10.1016/j.jbc.2021.101233

**Published:** 2021-09-23

**Authors:** Shivaksh Ahluwalia, Divya Choudhary, Purnima Tyagi, Vijay Kumar, Perumal Vivekanandan

**Affiliations:** 1 Kusuma School of Biological Sciences, Indian Institute of Technology Delhi, New Delhi, India; 2 Department of Chemical Engineering, Indian Institute of Technology Delhi, New Delhi, India; 3 Department of Molecular and Cellular Medicine, Institute of Liver and Biliary sciences, New Delhi, India

**Keywords:** vitamin D, hepatitis B virus, gene regulation, nuclear receptor, cell signaling, viral transcription, antiviral agent, vitamin D receptor, vitamin D response element, HBV core promoter, CHB, chronic HBV, ChIP, chromatin immunoprecipitation, DR, direct repeat, 6-FAM, 6-carboxyfluorescein, HBeAg, hepatitis B “e” antigen, HBsAg, hepatitis B surface antigen, HBV, hepatitis B virus, HCC, hepatocellular carcinoma, IVT, *in vitro* translated, NR, nuclear receptor, NTCP, sodium taurocholate cotransporting polypeptide, pcRNA, precore RNA, pgRNA, pregenomic RNA, qPCR, quantitative PCR, RXR, retinoid X receptor, VDR, vitamin D receptor, VDRE, vitamin D response element

## Abstract

Clinical and epidemiological studies support a role for vitamin D in suppressing hepatitis B virus (HBV). This antiviral role of vitamin D is widely attributed to vitamin D receptor (VDR)/retinoid X receptor–mediated regulation of host immunomodulatory genes through vitamin D response elements (VDREs) in their promoters. Here, we investigated the ability of calcitriol (1α,25-dihydroxyvitamin D3, metabolically activated vitamin D) to directly regulate HBV activity through this signaling pathway. We observed that calcitriol selectively inhibited only the HBV core promoter without affecting the HBV-PreS1, HBV-PreS2/S, or HBx promoters. We then identified a VDRE cluster in the HBV core promoter that is highly conserved across most HBV genotypes. Disruption of this VDRE cluster abrogated calcitriol-mediated suppression of the HBV core promoter. Furthermore, we showed that VDR interacts directly with the VDRE cluster in the HBV core promoter independent of retinoid X receptor. This demonstrates that calcitriol inhibits HBV core promoter activity through a noncanonical calcitriol-activated VDR pathway. Finally, we observed that calcitriol suppressed expression of the canonical HBV core promoter transcripts, pregenomic RNA, and precore RNA in multiple HBV cell culture models. In addition, calcitriol inhibited the secretion of hepatitis B “e” antigen and hepatitis B surface antigen (HBV-encoded proteins linked to poor disease prognosis), without affecting virion secretion. Our findings identify VDR as a novel regulator of HBV core promoter activity and also explain at least in part the correlation of vitamin D levels to HBV activity observed in clinical studies. Furthermore, this study has implications on the potential use of vitamin D along with anti-HBV therapies, and lays the groundwork for studies on vitamin D-mediated regulation of viruses through VDREs in virus promoters.

It is estimated that 2 billion people have been infected with hepatitis B virus (HBV) at some point in their lifetime, which has led to more than 350 million chronic HBV (CHB) infections worldwide (1). CHB leads to depletion of liver function and is a leading cause of hepatocellular carcinoma (HCC) (2). Hence, it is important to understand mechanisms regulating virus activity, so as to develop interventions to improve prognosis (3). Serum vitamin D level has been inversely linked to HBV activity in numerous clinical and epidemiological studies; however, the role of vitamin D in HBV biology is not well understood (4). In this study, we explore the ability of vitamin D to regulate HBV activity.

HBV is an enveloped small DNA virus belonging to the Hepadnaviridae family. Its 3.2-kb genome has four major promoters, which interact with host-encoded and HBV-encoded transcription factors to regulate HBV transcripts (5). A graphical illustration of the HBV genome depicting the relative position of the HBV promoters and their canonical transcripts has been provided (Fig. S1). The PreS1/S and PreS2 promoters yield two transcripts that are translated into surface proteins, which constitute the viral envelope. The HBx promoter regulates the production of the oncogenic HBx protein, and the HBV core promoter regulates two transcripts: the precore RNA (pcRNA) and the pregenomic RNA (pgRNA). The HBV pgRNA represents the full-genetic template of the virus that is packaged into the capsid and later reverse transcribed. The HBV pcRNA encodes the hepatitis B “e” antigen (HBeAg), which is secreted from infected hepatocytes (6). In addition to HBeAg, the HBV genome encodes another secretory protein, hepatitis B surface antigen ([HBsAg]; an HBV surface protein). These secretory proteins are primary markers used for diagnosis of HBV infection, and their presence is linked to poor disease prognosis (6, 7, 8, 9, 10, 11, 12, 13).

Vitamin D can regulate genes involved in vital cellular processes, such as mineral homeostasis, cell cycle regulation, and immunomodulation, through its metabolically active form known as calcitriol or 1α,25-dihydroxyvitamin D3. Calcitriol binds and activates a nuclear receptor (NR) known as vitamin D receptor (VDR). VDR can activate or repress transcription by binding DNA motifs known as vitamin D response elements (VDREs) at proximal or distal sites from the transcription start site (14). VDREs are usually composed of two hexameric core sequences (direct repeats [DRs]) separated by three nucleotides (DR3-type VDREs) (14, 15). Activated VDR usually heterodimerizes with another NR, retinoid X receptor (RXR), at VDREs to activate gene expression (14). Deviations from conventionally observed events in the VDR pathway are associated with negative regulation of the target gene. For instance, VDR has been shown to bind to VDREs in the absence of RXR to suppress transcription (16, 17, 18, 19).

Vitamin D deficiency in CHB is linked to increased viral replication (20, 21), poor disease prognosis, and progression to HCC (22, 23). Patients with CHB having sufficient vitamin D levels show better response to anti-HBV therapy, as observed by an improved virological response to nucleos(t)ide analogs (24). Furthermore, aberrations in the *VDR* gene, specifically VDR *FokI* polymorphisms, are associated with increased susceptibility to HBV infection and an increased risk of HCC (25, 26). Taken together, these clinical findings strongly support the role of vitamin D and its induced VDR pathway in regulating HBV activity and HBV-related liver disease. These observations have been primarily attributed to the ability of vitamin D to improve the innate and adaptive immune response of the host (27, 28). The presence of VDREs in the HBV genome and the regulation of its promoters directly through the VDR signaling pathway has not been explored. Here, we hypothesized that vitamin D can regulate HBV promoter activity directly through its signaling pathway, therefore potentially altering transcription, translation of viral proteins, and ultimately HBV replication. Our hypothesis is supported by numerous studies that have shown that other members of the NR superfamily, including peroxisome proliferator–activated receptor α, RXRα, farnesoid X receptor alpha, hepatocyte nuclear factor-4, and testicular receptor 4 can directly bind and regulate transcription from the HBV genome (29, 30, 31, 32). Furthermore, VDR has been previously suggested to directly bind and alter viral promoter activity in HIV-1 (33).

In this study, we tested the ability of calcitriol to regulate HBV promoters directly. We demonstrated that calcitriol negatively regulates only the HBV core promoter. We then screened the core promoter for the presence of functional VDREs and tested their role in the calcitriol-mediated inhibition of the core promoter. We further used binding assays to better understand the mechanism by which calcitriol and its target receptors inhibit the core promoter. Finally, we studied the effect of calcitriol in context of the whole-HBV genome, for its ability to regulate HBV core promoter transcripts (pgRNA and pcRNA), HBV secretory proteins (HBsAg and HBeAg), and HBV virion secretion in different HBV cell culture models.

## Results

### HBV core promoter is selectively suppressed by calcitriol

Luciferase reporter assays were performed to test the effect of calcitriol on the activity of each of the four HBV promoters in three hepatic cell lines: HepG2, Huh7, and HepG2.2.15. Before conducting these experiments, we wanted to ensure these cell lines respond to the vitamin D-activated VDR pathway. For this purpose, we studied the expression of CYP24A1, a host gene that is known to be transactivated by VDRE–VDR/RXR interactions in its promoter (34, 35, 36). We observed a significant increase in the expression of CYP24A1 mRNA by quantitative PCR (qPCR) in all the three hepatic cell lines in the presence of 10 nM calcitriol, confirming that the vitamin D signaling pathway is active in all the three cell lines used in this study (Fig. S2).

**Figure 1:**
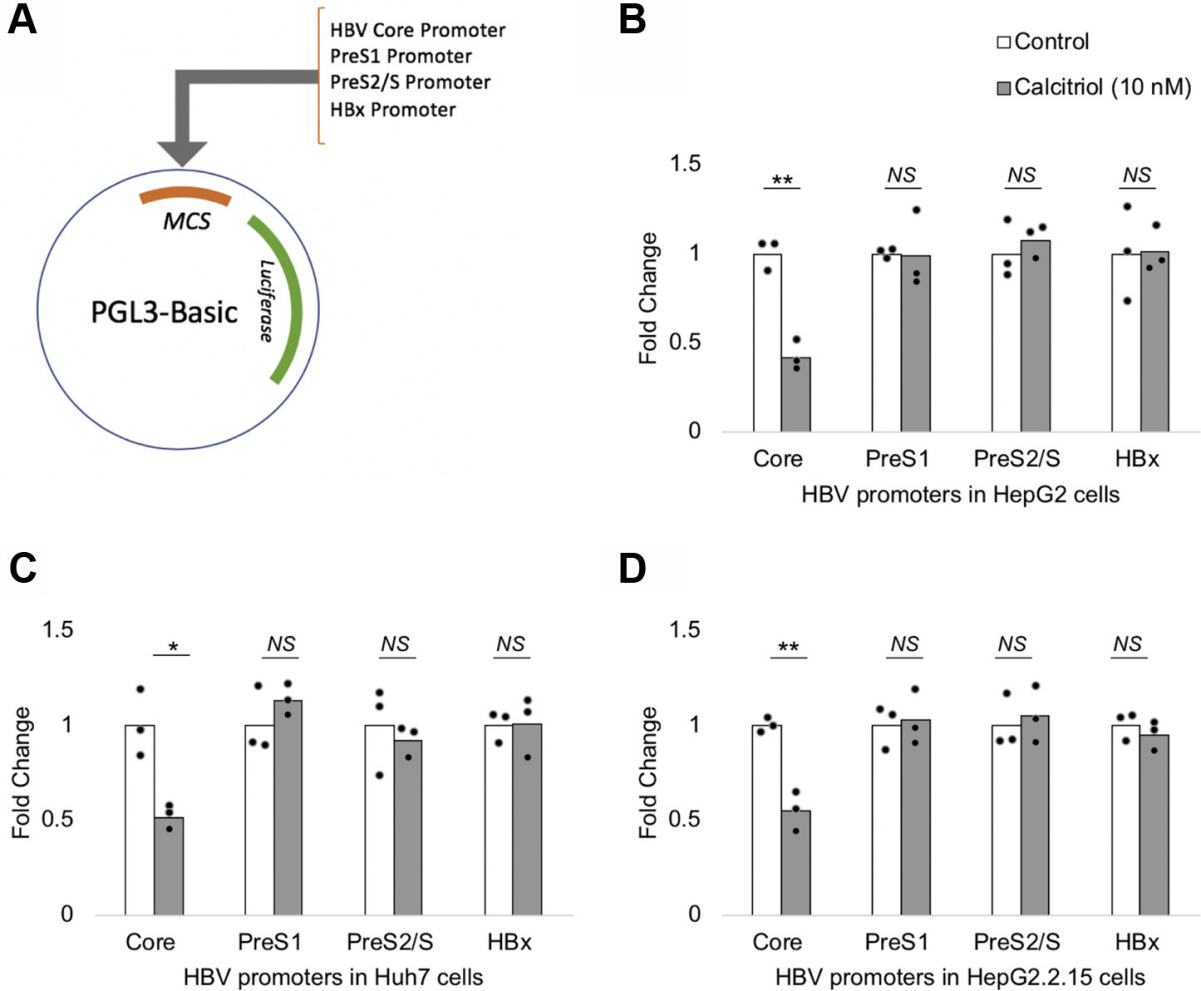
**Calcitriol selectively suppresses the HBV core promoter in luciferase assays.***A*, each of the four HBV promoters (HBV-core, PreS1, PreS2/S, and HBx promoter) were cloned upstream of the luciferase reporter gene in the PGL3-basic construct. The activity of each of the promoters was tested in the presence of vehicle control (without calcitriol) or 10 nM calcitriol in hepatic cell lines, (*B*) HepG2, (*C*) Huh7, and (*D*) HepG2.2.15 by luciferase assay, 24 h after transfection. HBV core promoter activity was significantly supressed, whereas the activity of the remaining HBV promoters was unaffected in the presence of the ligand. All data are means ± SD for three independent experiments (n = 3). ∗*p* < 0.05, ∗∗*p* < 0.01, and NS (analyzed by paired Student's *t* test). HBV, hepatitis B virus; NS, not significant.

Each of the four HBV promoters (*i.e.*, HBV core promoter, PreS1 promoter, PreS2/S promoter, and HBx promoter) were cloned separately into the PGL3-basic construct, upstream of the luciferase reporter gene (Fig. 1*A*). The activity of each of the four HBV promoters was assessed using luciferase reporter assays. The addition of 10 nM calcitriol led to a significant reduction in the HBV core promoter activity in all the three cell lines; whereas the other HBV promoters were not affected (Fig. 1, *B*–*D*). It should be noted that the HepG2.2.15 cells contains two head-to-tail dimers of the HBV type “D” genome (37). Therefore, the HBV transcripts and proteins constitutively expressed in HepG2.2.15 did not affect the calcitriol-mediated inhibition of the HBV core promoter in reporter assays.

### The HBV core promoter contains a highly conserved cluster of putative VDREs

**Figure 2:**
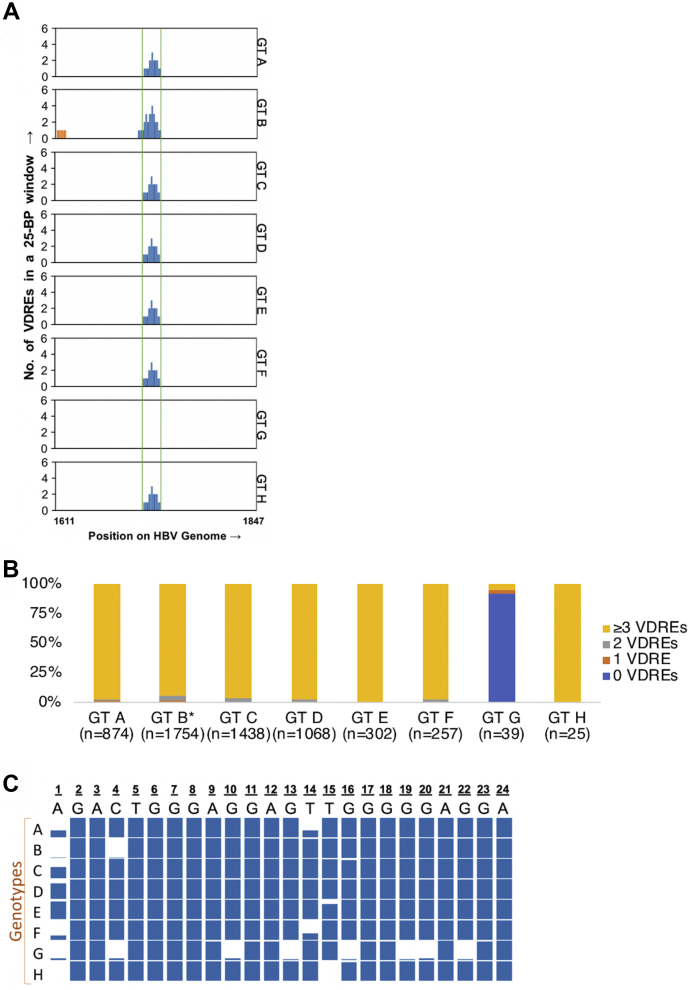
**Identification of a VDRE cluster in the HBV core promoter that is conserved across most HBV genotypes.***A*, in silico analysis identified a cluster of overlapping VDREs on the sense strand (*blue bars*) in the HBV core promoter (1724–1748 in genotype “D,” highlighted with *green box*) in seven HBV genotypes (GT A, GT B, GT C, GT D, GT E, GT F, and GT H; nucleotide numbering may vary subtly across HBV genotypes). HBV genotype “G” was the exception with no VDREs in this region. HBV genotype “B” in addition has one VDRE on the antisense strand (*orange bar*). *B*, graphical representation of conservation of the HBV VDRE cluster within genotypes. The HBV core promoter sequence of genotypes A to H in the HBV database (n = 5757) was analyzed for the presence of VDREs. More than 94% of all sequences of seven HBV genotypes (GT A, GT B, GT C, GT D, GT E, GT F, and GT H) had three or more VDREs. Most of the sequences from HBV genotype “G” had no VDREs. *C*, majority of the positions of the 24-nucleotide region corresponding to the HBV VDRE cluster is highly conserved across HBV genotypes. All sequences from genotypes A to H in the HBV database (n = 5757) were analyzed for conservation using the HBV VDRE cluster of HBV genotype “D” (sequence on *top*). The height of each bar corresponds to the percentage of sequences with a conserved nucleotide at a given position when using the VDRE cluster from HBV genotype “D” as the reference sequence. This region was highly conserved across most HBV genotypes, with the exception of genotype “G”. ∗Majority (>85%) of the sequences in genotype “B” had an additional VDRE cluster (*i.e.*, four VDREs in the cluster instead of three). HBV, hepatitis B virus; VDRE, vitamin D response element.

Eight consensus sequences representing each of the HBV genotypes (A–H) were generated from the sequences (n = 5757) available in the HBV database (https://hbvdb.lyon.inserm.fr/HBVdb/HBVdbIndex) (38). We screened for DR-3 type VDREs in the sense and antisense strand of HBV core promoter in these consensus sequences, using the criterion described in the Experimental procedures section. The results have been graphically represented as the number of putative VDREs in a sliding window of 25 base pairs along the consensus sequence of the HBV core promoter for each genotype. A peak corresponding to a cluster of three or more putative VDREs was identified within upstream regulatory region of the HBV core promoter in all HBV genotypes, with the exception of genotype “G”, which had no VDREs (Fig. 2*A*). Only genotype “B” had an additional putative VDRE on its antisense strand.

All experiments in this study were carried out with HBV genotype “D” (HBV serotype *ayw*). It contains a total of three putative VDREs clustered in a window of 24 nucleotides (from 1724 to 1748; numerical coordinates on HBV genome as per GenBank Sequence V01460.1). The position and sequence of the VDREs in HBV genotype “D” have been described in Table S1.

Analysis of all genotype “D” sequences in the HBV database (n = 1068) revealed that the presence of a VDRE cluster in the core promoter was consistent, with over 95% of the sequences having at least three VDREs clustered together in the core promoter. In fact, >94% of all sequences (n = 5757) of all HBV genotypes (A, B, C, D, E, F, and H) had three or more VDREs clustered together in the core promoter, with the exception genotype “G” in which majority of sequences did not contain any VDREs (Fig. 2*B*). Further analysis of the 24-nucleotide sequence carrying the VDRE cluster showed that it was highly conserved at each position across HBV genotypes A to H, with the exception of genotype “G” (Fig. 2*C*). This is consistent with our findings using the consensus sequence of genotypes A to H (Fig. 2*A*) and those with each sequence within genotypes A to H (Fig. 2*B*), where we observe VDREs are conserved across HBV genotypes other than genotype “G.” Taken together, these findings show that VDREs are consistently observed in the HBV core promoter, and the region in which they cluster is highly conserved.

### Disrupting VDREs in the HBV core promoter abrogates calcitriol-mediated suppression

**Figure 3:**
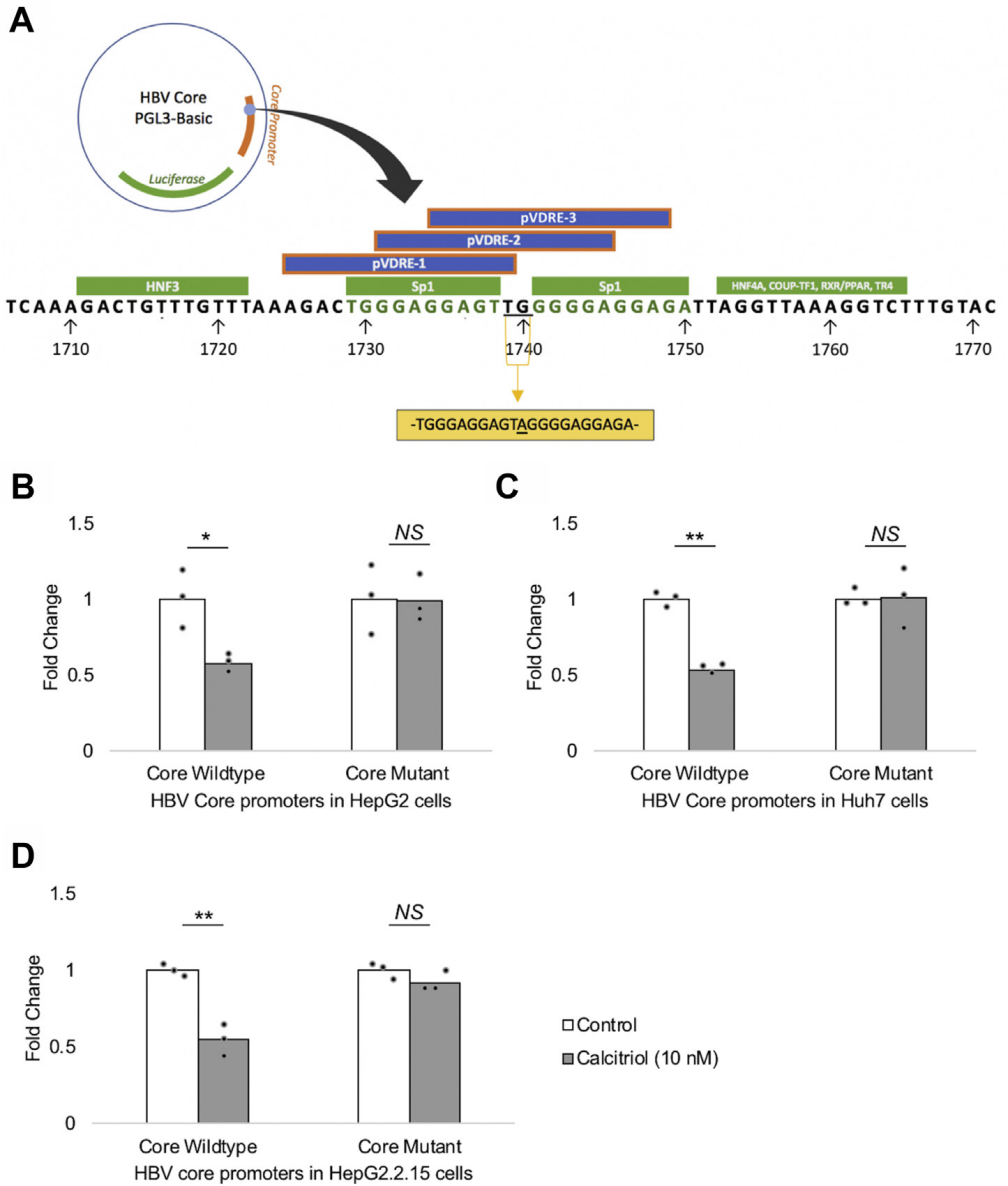
**Mutations disrupting the HBV VDRE cluster abrogates calcitriol-mediated suppression of the HBV core promoter.***A*, a region of the HBV core promoter on which various nuclear receptor response elements (NRREs) have been mapped (*green boxes*), along with the identified overlapping cluster of putative VDREs (pVDREs, *blue boxes* bordered with *orange*). The high degree of overlap of the VDREs with other NRREs permits the alteration of only two base pairs (*underlined*). Mutations at these two positions (the *yellow box* shows the mutated core promoter sequence), performed as described in the Experimental procedures section, disrupt the sequence of the three VDREs in the cluster. Luciferase assays were performed to test the response of this mutated HBV core promoter (core mutant; with VDREs disrupted) as compared with the wildtype core promoter (core wildtype) in the presence of 10 nM calcitriol or vehicle control (without calcitriol) in (*B*) HepG2, (*C*) Huh7, and (*D*) HepG2.2.15 cells. Mutations disrupting the VDRE cluster abrogate the response of HBV core promoter to vitamin D signaling. All data are means ± SD for three independent experiments (n = 3). ∗*p* < 0.05, ∗∗*p* < 0.01, and NS (analyzed by paired Student's *t* test). COUP-TF1, chicken ovalbumin upstream promoter-transcription factor 1; HBV, hepatitis B virus; HNF3, hepatocyte nuclear factor 3; HNF4A, hepatocyte nuclear factor 4 alpha; NS, not significant; PPAR, proliferator–activated receptor; RXR, retinoid X receptor; TR4, testicular receptor 4; VDRE, vitamin D response element.

We then wanted to test whether calcitriol-mediated suppression of the HBV core promoter occurred through the identified VDREs. To that end, we created mutations in the HBV core promoter to disrupt VDREs, while ensuring motifs binding other transcription factors were unaltered. Detailed analysis revealed that the identified VDRE cluster was in close proximity to numerous important nuclear receptor response elements and overlapped with two Sp1-binding sites (Fig. 3*A*) (29, 32, 39, 40, 41). Mutating Sp1 sites have been previously shown to almost completely inhibit transcription from the HBV core promoter, hence this restricted the number of bases that could be altered to two (1739 and 1740; underlined bases in Fig. 3*A*) for the purpose of disrupting VDREs in the cluster (41) (please see the Experimental procedures section for details on the mutations disrupting VDREs).

The activity of mutated HBV core promoter was compared with that of the wildtype HBV core promoter in the presence or the absence of calcitriol in HepG2, Huh7, and HepG2.2.15 cell lines using luciferase assays. As observed earlier, expression of the wildtype HBV core promoter (core wildtype) was significantly reduced in the presence of the ligand. However, there was no significant change in the activity of the mutated HBV core promoter (core mutant) in the presence of calcitriol in the three hepatic cell lines (Fig. 3, *B*–*D*). These findings showed that disrupting the identified VDREs abrogated the calcitriol-mediated suppression of the HBV core promoter.

In silico analysis showed that the HBV genotype “G” did not contain the identified VDREs (Fig. 2*A*). Hence, we wanted to test the effect of calcitriol on the core promoter of this naturally occurring HBV variant lacking VDREs. The core promoter activity of HBV genotype “G” remained unaffected in the presence of calcitriol in luciferase reporter assays in the three hepatic cell lines; thus further supporting our findings that calcitriol acts through the identified VDRE cluster to regulate HBV core promoter activity (Fig. S3, *A–C*).

### VDR binds to the identified HBV VDRE cluster to regulate HBV core promoter activity

Having demonstrated that calcitriol acts through the identified HBV VDREs to suppress core promoter activity, we went on to perform gel shift assays to investigate whether VDR–RXR interacts directly with the HBV VDRE cluster. A fragment of the Rat pit-1 promoter carrying an established VDRE known to bind VDR and RXR was used as a procedural positive control for the EMSA (Fig. S4) (42).

**Figure 4:**
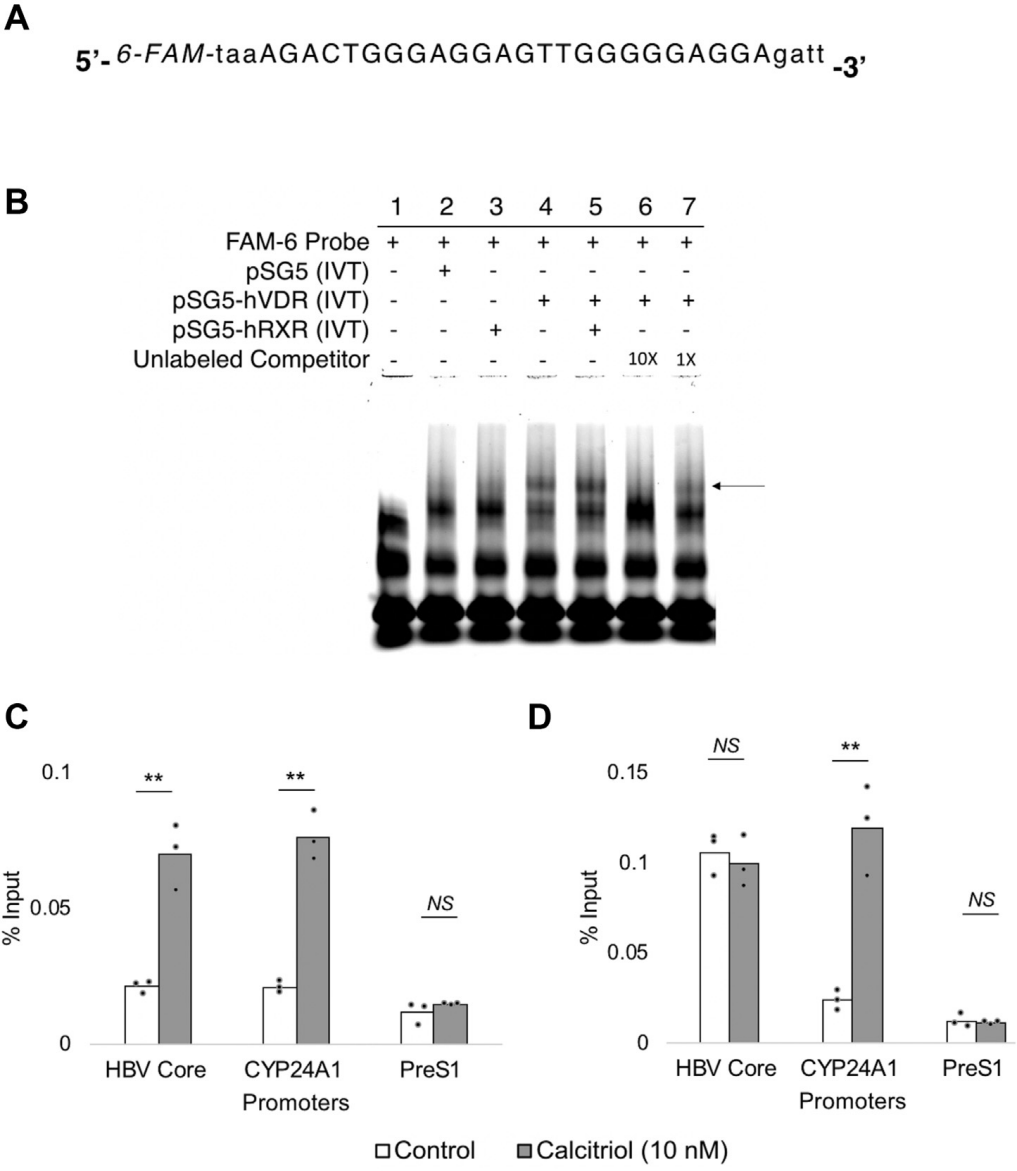
**VDR binds to VDREs in the HBV core promoter independent of RXR.***A*, a fragment of the HBV core promoter carrying the identified VDRE cluster (*capitalized*) labeled with the 6-FAM fluorophore at the 5′ end of the probe. *B*, EMSA was performed using *in vitro* translated (IVT) VDR and RXR as detailed in the Experimental procedures section. Briefly, the 6-FAM-labeled probe was incubated with VDR and/or RXR and unlabeled competitor oligonucleotides when required and resolved on a native 8% polyacrylamide gel. VDR interacts with the VDREs in the HBV core promoter in the absence of RXR, as observed by the presence of the indicated band (see *arrow*) in *lane 4*. The addition of unlabeled competitor oligonucleotides reduced the complex formed in a concentration-dependent manner, demonstrating the specificity of the binding reaction (see *lanes 6* and *7*). Representative gel image from one of three separate experiments is shown. *C*, ChIP-quantitative PCR (qPCR) using anti-VDR antibody confirmed the increased binding of VDR at the HBV core promoter in HepG2 cells transfected with 1.3× HBV-genome construct in the presence of 10 nM calcitriol for 24 h. *D*, whereas, no enrichment of RXR was observed at the core promoter when ChIP-qPCR was performed with anti-RXR antibody in the presence of the ligand in similar conditions. The CYP24A1 promoter having established VDREs was used as a positive control, whereas the HBV PreS1 promoter having no putative VDREs was used as a negative control. All data are means ± SD for three independent experiments (n = 3). ∗*p* < 0.05, ∗∗*p* < 0.01, and NS (analyzed by paired Student's *t* test). 6-FAM, 6-carboxyfluorescein; HBV, hepatitis B virus; NS, not significant; RXR, retinoid X receptor; VDR, vitamin D receptor; VDRE, vitamin D response element.

Fluorescently labeled (6-carboxyfluorescein [6-FAM]) double-stranded probes carrying the VDRE cluster of the HBV core promoter (Fig. 4*A*) were incubated with *in vitro* translated (IVT) VDR and/or RXR to study their binding. We observed that VDR interacts with the HBV VDRE cluster independent of RXR (*lane 4*, Fig. 4*B*). RXR alone could not interact with the HBV VDRE cluster (*lane 3*, Fig. 4*B*). Furthermore, addition of unlabeled double-stranded oligonucleotides caused a concentration-dependent reduction in the complex formed, confirming that the observed band corresponds specifically to the VDR-probe interaction (*lanes 6* and *7*, Fig. 4*B*).

We also performed chromatin immunoprecipitation (ChIP) to assess the interaction of VDR and RXR with the HBV core promoter in HepG2 cells transfected with the greater-than-genome-length HBV construct in the presence or the absence of calcitriol. The chromatin from these cells was extracted, and genetic material bound to VDR or RXR was pulled down using their respective antibodies, followed by its quantitation by qPCR (see the Experimental procedures section for details). We observed approximately a 3.5-fold increase in binding of VDR to the HBV core promoter in the presence of calcitriol (Fig. 4*C*). However, no significant enrichment of RXR was observed at the HBV core promoter in the presence of the ligand, thereby further supporting our finding that VDR interacts with the HBV core promoter independent of RXR (Fig. 4*D*). The CYP24A1 promoter contains established VDREs known to bind VDR and RXR and hence served as a positive control (34, 35, 36). We observed an increased binding of both VDR and RXR at the CYP24A1 promoter in the presence of calcitriol, demonstrating that the canonical vitamin D signaling pathway is active in these cells. The HBV preS1 promoter did not have any putative VDREs and served as a negative control for this experiment. No significant enrichment of VDR and RXR was observed at the PreS1 promoter. Taken together, these results indicate that VDR binds to the HBV VDRE cluster in a RXR-independent manner to suppress HBV core promoter activity. As observed in the HBV core promoter, noncanonical RXR-independent binding of VDR has been previously shown to suppress promoter activity (16, 17, 18, 19).

### Calcitriol suppresses transcription and translation from the HBV core promoter in the full-length HBV genome

**Figure 5:**
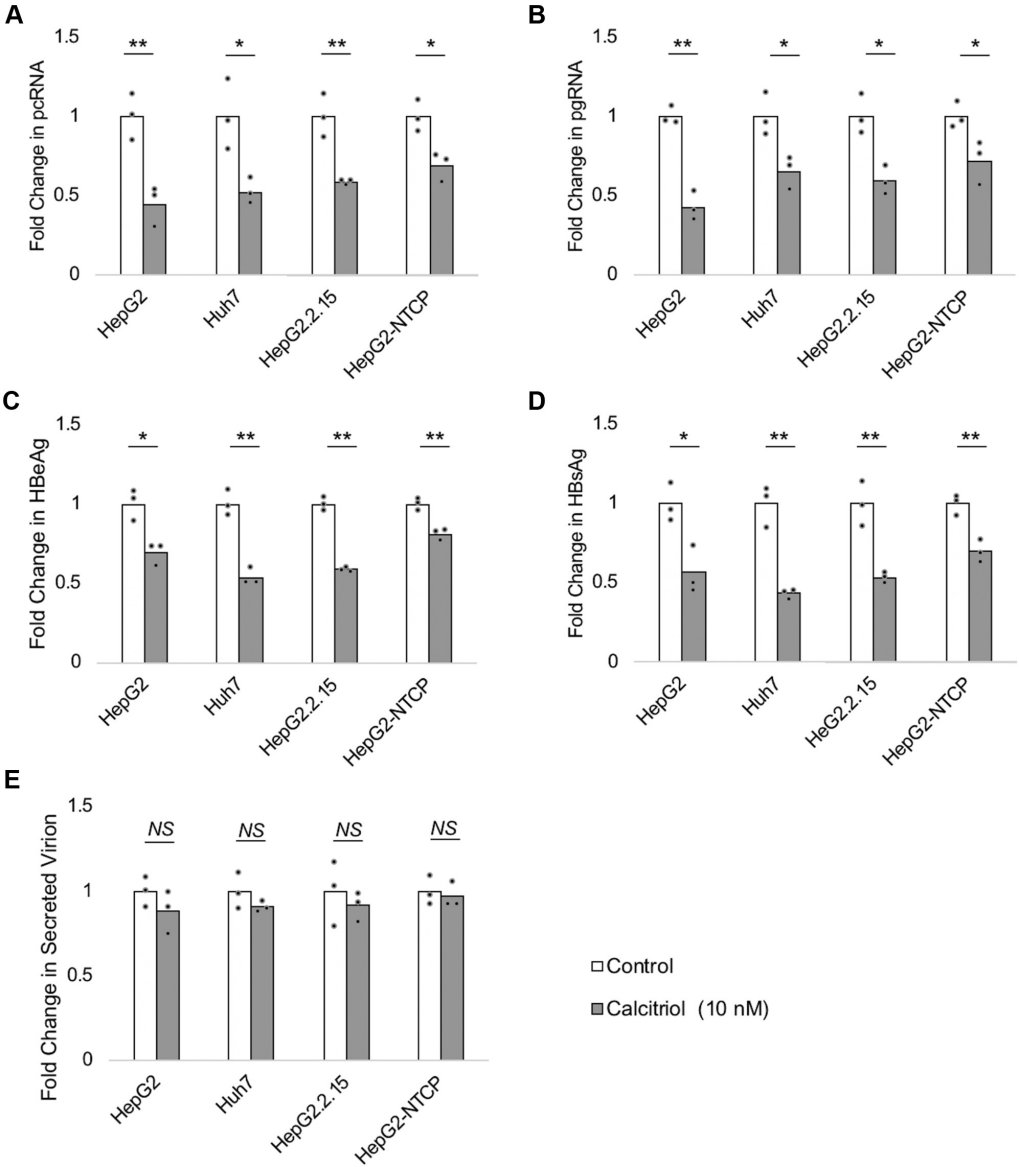
**Calcitriol inhibits HBV core promoter transcripts, HBV secretory proteins, but not HBV virion secretion.** The 1.3×-HBV genome construct was transfected in HepG2 and Huh7 cells, whereas HepG2.2.15 stably expresses HBV genome. In the HBV infection model, HBV particles derived from HepG2.2.15 cells were used to infect HepG2-NTCP cells as described in the Experimental procedures section. Vehicle control (without calcitriol) or 10 nM calcitriol was added immediately after transfection or infection, and samples were processed as described in the Experimental procedures section. RNA isolation and quantitation by quantitative PCR (qPCR) shows that (*A*) HBV pcRNA and (*B*) HBV pgRNA are suppressed in the presence of calcitriol. Quantitation of HBV secretory proteins by ELISA demonstrates that calcitriol negatively regulates (*C*) HBeAg and (*D*) HBsAg. *E*, finally, HBV virion secreted in supernatant was captured 72 h after ligand treatment, and its DNA was quantitated by qPCR. The virion secretion in the presence of calcitriol was marginally reduced in all HBV cell culture models (up to 12%) *in vitro*, though the observation was not statistically significant in any model. All data are means ± SD for three independent experiments (n = 3). ∗*p* < 0.05, ∗∗*p* < 0.01, and NS (analyzed by paired Student's *t* test). HBeAg, hepatitis B “e” antigen; HBsAg, hepatitis B surface antigen; HBV, hepatitis B virus; NS, not significant; NTCP, sodium taurocholate cotransporting polypeptide; pcRNA, precore RNA; pgRNA, pregenomic RNA.

The HBV core promoter gives rise to two transcripts, pgRNA and pcRNA. They have been observed to express coordinately as well as distinctly from each other. Therefore, the HBV core promoter has been considered to function as two independent promoters regulating two distinct transcripts (39). Hence, we assessed the calcitriol-mediated regulation of both pgRNA and pcRNA in HepG2, Huh7, and HepG2.2.15 cell lines, as well as in HepG2–sodium taurocholate cotransporting polypeptide (NTCP) cells infected with HBV. It should be noted that these cell lines represent three different HBV cell culture models: (i) greater-than-genome-length HBV transfection model (in HepG2 and Huh7 cells), (ii) stably integrated HBV-genome model (HepG2.2.15 cells), and (iii) HBV infection model, in which HepG2.2.15-derived HBV was used to infect HepG2–NTCP cells. The addition of calcitriol led to a significant reduction in pcRNA (Fig. 5*A*) and pgRNA (Fig. 5*B*) levels in all three models. We further quantitated the HBeAg secreted using ELISA and observed a similar reduction in this translated product of pcRNA in the presence of calcitriol (Fig. 5*C*). We also observed a similar reduction in HBsAg secretion by ELISA in the presence of the ligand (Fig. 5*D*). Taken together, these results further support that calcitriol negatively regulates HBV core promoter activity, as observed by reduction of HBV pcRNA and pgRNA transcripts in three different HBV-cell culture models. We also show that calcitriol inhibits the production of secretory HBeAg and HBsAg. It should be noted that both these secretory proteins have been linked to poor disease prognosis in HBV infection (8, 9, 10, 11, 12, 13).

### Calcitriol does not lead to a significant reduction in virion secretion

Clinical studies indicate that vitamin D inhibits HBV activity (20, 21). Here, we tested the effect of calcitriol on HBV virion secretion *in vitro*. We captured secreted virions from the supernatant of cells, (i) either transfected with greater-than-genome-length constructs of HBV (HepG2 and Huh7), (ii) having an integrated HBV genome (HepG2.2.15), or (iii) infected with HBV (HepG2–NTCP) in the presence of calcitriol, as described previously. The DNA from the captured virions was isolated and quantitated by qPCR. We observed that virion secretion in each of the models was marginally reduced (by up to 12%) in the presence of calcitriol, but this reduction was not statistically significant in any of the models (Fig. 5*E*).

## Discussion

The antiviral mechanisms of vitamin D are poorly understood and generally attributed to its ability to regulate host immunomodulatory genes by vitamin D-activated VDR pathways, thereby strengthening host immune response (27, 28, 43, 44). However, in this study, we demonstrate that vitamin D signaling acts directly through a VDRE cluster in the HBV core promoter suppressing key HBV transcripts and HBV proteins. This study hence conceptually advances our current understanding of the antiviral role of vitamin D and highlights the ability of VDR to bind to VDREs in the virus genome to suppress virus activity.

We demonstrated that calcitriol negatively regulated HBV core promoter activity in three hepatic cell lines (Fig. 1), whereas the other HBV promoters were unaffected. We then screened the HBV core promoter for DR3-type VDREs *in silico* and identified a conserved cluster of three overlapping VDREs (1724–1748) in the upstream regulatory region of the HBV core promoter. Studies have identified HBV core promoter mutants having unique clinical pathogenesis. Some of these mutations, for instance, that at 1741, overlap with the identified VDREs (45). Studying VDR–VDRE interactions in relevant core promoter mutants may help us better understand the basis of differences in the unique clinical outcomes associated with these variants.

The VDREs in the HBV core promoter were highly conserved across HBV genotypes “A to H”, with the exception of genotype “G”, which had no VDREs (Fig. 2*A*). Our findings suggest that the absence of VDREs (occurring naturally in HBV genotype “G” core promoter; Fig. S3) or the disruption of VDREs (by mutations introduced in HBV genotype “D”; Fig. 3) in the HBV core promoter render it nonresponsive to vitamin D signaling. These results confirm that vitamin D regulates the HBV core promoter through the identified VDRE cluster in the HBV genome and also validates our in silico method for identifying VDREs. The ability of vitamin D to inhibit viral genes directly through VDREs in the virus genome has not been reported previously.

A complex network of host nuclear factors regulates HBV core promoter activity. Some of these factors are ubiquitous, such as Sp1 and TATA-binding protein, whereas others, such as hepatocyte nuclear factor-3 and hepatocyte nuclear factor-4, are specifically enriched in the liver (45, 46). In this study, we identified VDR, another ubiquitous member of this superfamily of NRs, which binds and regulates the HBV core promoter. Conventionally, VDR heterodimerizes with RXR to bind DR3-type VDREs to activate gene expression. In contrast, we demonstrate that ligand-activated VDR binds to the VDRE cluster in the HBV core promoter independent of RXR (Fig. 4, *B*–*D*). This noncanonical RXR-independent binding of VDR to VDREs has previously been reported to suppress gene expression (16, 17, 18, 19). In keeping with these findings, we also observe that the RXR-independent binding of VDR to the HBV core promoter leads to its inhibition.

The addition of calcitriol led to a significant decrease in transcription of pgRNA and pcRNA (Fig. 5, *A* and *B*). Furthermore, calcitriol significantly inhibited HBeAg and HBsAg secretion in the 1.3× HBV genome transfection model, integrated HBV-genome model, and *in vitro* HBV infection model (Fig. 5, *C* and *D*); this is keeping with previous studies that have linked core promoter activity to HBeAg and HBsAg levels (41, 47, 48, 49). Clinical studies have linked higher levels of HBV secretory proteins with increased risk of liver fibrosis, progression to HCC, and poorer clinical outcomes (8, 9, 10, 11, 12, 13). Numerous studies have identified cellular mechanisms by which these HBV secretory proteins can lead to the development of HCC (50, 51, 52, 53, 54). In addition, reduction in HBsAg levels and loss of HBeAg have been used as therapeutic end points (55, 56). The pathogenic role of HBeAg and HBsAg highlights the clinical significance of vitamin D-mediated suppression of these HBV proteins and merits further investigation on supplementation of this micronutrient along with anti-HBV therapies to improve prognosis in CHB.

In this study, we observe a marginal reduction in virion secretion in the presence of calcitriol; however, this reduction was not statistically significant (Fig. 5*E*). HBV pgRNA serves as a vital replicative intermediate in the HBV lifecycle; hence, its levels are directly linked to HBV replication, thereby virion secretion. In contrast, HBeAg has been shown to inhibit HBV replicative activity in cell culture models (57, 58). The reduced HBeAg levels in the presence of vitamin D may augment to HBV replication, enhancing virion production and secretion; thus masking the impact of decreased HBV pgRNA levels on virion secretion. Furthermore, numerous host genes have been shown to regulate HBV transcription and translation. Many host genes are involved in important stages of the HBV lifecycle, such as viral particle assembly and secretion (5). Vitamin D and its target receptors have been previously shown to extensively alter the cellular transcriptome and regulate numerous cellular processes (59). Vitamin D may affect crucial host genes involved in the HBV lifecycle. For instance, cdc2-like kinase is involved in core protein phosphorylation, a process that is essential for virion assembly (60). Studies have shown that calcitriol suppresses cdc2-like kinase activity and therefore can potentially inhibit HBV assembly (61, 62). This may at least in part explain the lack of significant inhibition of virion secretion *in vitro.*

The liver plays a pivotal role in metabolism in the body; hence, hepatocytes have the ability to quickly respond to the availability of nutrients and micronutrients by altering its gene expression profile through various transcription factors. Shlomai and Shaul (63) proposed HBV to be a model of a “metabolovirus”; they suggested that HBV couples its transcriptional and replicative activity to nutritional cues using these transcription factors. For instance, metabolic genes such as PEPCK and G6Pase activated during gluconeogenesis, can also enhance HBV replication. Farnesoid X receptor alpha is another NR that can regulate transcriptional activity of the HBV core promoter, and its expression is enhanced during starvation (31). We propose micronutrients should also be added to this model for their ability to regulate HBV activity. Studies have implicated the potential role of vitamin A (through RXRα) (30, 64) and vitamin E in regulating HBV replication (65, 66). In this study, we show that vitamin D is an important micronutrient that can regulate HBV activity. Taken together, these studies highlight the importance of nutrition in managing hepatitis B.

**Figure 6:**
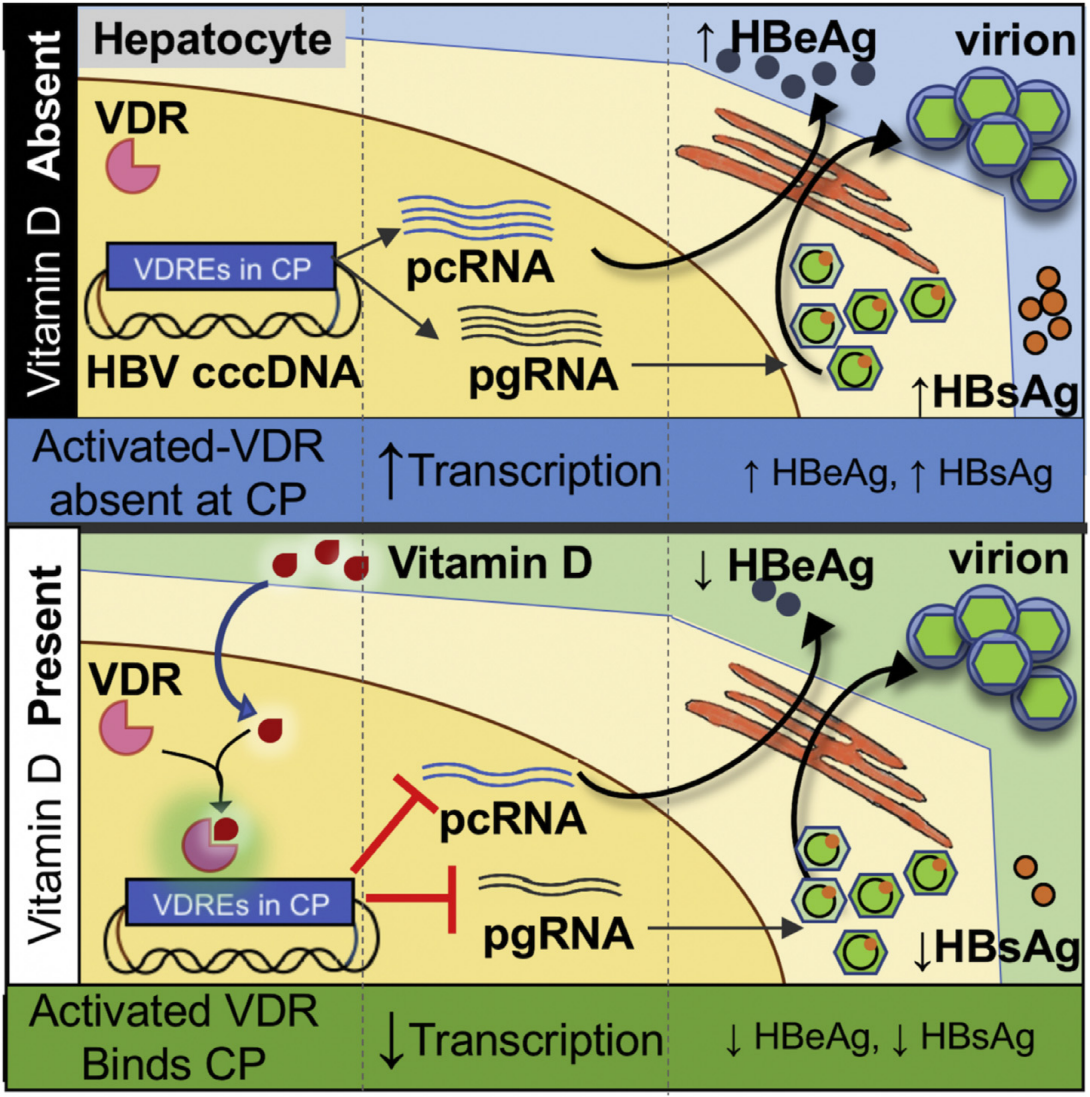
**Calcitriol inhibits HBV activity directlythroughthe vitamin D signaling pathway.** The HBV core promoter (CP) transcribes the pgRNA and pcRNA. The pgRNA serves as the genetic template for the virus and is packaged into virus particles before secretion. The pcRNA is translated to the secretory HBeAg and then secreted *via* the endoplasmic reticulum (ER) (*upper panel*, see *left* to *right*). Calcitriol (metabolically activated vitamin D) binds and activates its nuclear receptor, VDR (see *lower panel*). We identified a VDRE cluster in the HBV core promoter. Calcitriol-activated VDR interacts with the identified VDREs in the HBV core promoter independent of RXR, suppressing its activity. This inhibits transcription of pgRNA and pcRNA. HBeAg and HBsAg secretion is also inhibited in the presence of the ligand. Numerous studies have highlighted the pathogenic function of HBeAg and HBsAg; hence, the vitamin D-mediated inhibition of these secretory proteins may be of clinical relevance. We did not observe significant inhibition in HBV virion secretion in the presence of vitamin D. HBV, hepatitis B virus; HBeAg, hepatitis B “e” antigen; HBsAg, hepatitis B surface antigen; pcRNA, precore RNA; pgRNA, pregenomic RNA; VDR, vitamin D receptor; VDRE, vitamin D response element.

In summary, we identified functional VDREs within the HBV core promoter, which can directly bind VDR noncanonically in an RXR-independent manner leading to suppression of HBV activity in (a) reporter assays, (b) 1.3× HBV constructs in liver cells, (c) a hepatic cell line with stably integrated HBV genome, and (d) in an *in vitro* HBV infection model. Vitamin D signaling through VDREs in the HBV core promoter leads to a reduction in HBV transcripts (HBV pgRNA and HBV pcRNA) and HBV proteins (HBeAg and HBsAg) (Fig. 6). The identification of VDR as a novel host factor regulating HBV core promoter activity advances our current understanding of HBV–host interactions. Of note, the ability of vitamin D to reduce HBeAg and HBsAg levels suggests a potential role for this micronutrient as a supplement along with antiviral therapies for HBV. Most of our understanding of the antiviral role for vitamin D is limited to its ability to modulate host genes, primarily immune response genes. In contrast, this work sheds light on how vitamin D signaling may directly impact virus activity through VDREs present in virus genomes. Our results lay the groundwork for further studies on mapping of VDREs in virus genomes and understanding their biological role.

## Experimental procedures

### Cell culture, transfection, and calcitriol treatment

HepG2, Huh7, and HepG2.2.15 cells were grown in Dulbecco's modified Eagle's medium (Gibco) supplemented with 10% fetal bovine serum (Gibco), 2 mM l-glutamine (Himedia Laboratories Pvt Ltd), and penicillin–streptomycin solution (100 U/ml each; Gibco) at 37 °C and 5% CO_2_. Transfection was done using Lipofectamine 2000 (Invitrogen) as per the manufacturer's protocol. We added 10 nM of calcitriol (Sigma) dissolved in 95% ethanol or vehicle control (95% ethanol) to the medium immediately after transfection.

HepG2-hNTCP-C4 cells constitutively express the NTCP membrane transporter required for HBV internalization, thus making these cells susceptible to HBV infection. These cells were grown in Dulbecco's modified Eagle's medium/F-12 + GlutaMax (Gibco) supplemented with 10 mM Hepes (Gibco), 200 units/ml penicillin, 200 μg/ml streptomycin, 10% fetal bovine serum (Gibco), 50 μM hydrocortisone (Sigma), 5 μg/ml insulin (Gibco), and 400 μg/ml G418 (Himedia Laboratories Pvt Ltd; TC025), as described previously (67).

### HBV preparation and infection

HBV derived from HepG2.2.15 cells (genotype “D”) was used to infect HepG2-NTCP cells as described previously (67, 68). Briefly, media from HepG2.2.15 cells grown in T-175 flask were harvested every 3 days, cleared by centrifugation, and then precipitated with PEG8000 (Promega; V3011) and 2.3% NaCl. The precipitate containing HBV was washed and resuspended in media at 200-fold concentration, followed by quantitation of the HBV DNA by real-time PCR (see Table S2 for details on primers).

HepG2-hNTCP-C4 cells were infected in 6-well plates at 1 × 10^6^ genome equivalents/cell in infection media (complete media used for culturing HepG2-hNTCP-C4 cells described previously, supplemented with 4% PEG8000 and 2% dimethyl sulfoxide) for 24 h (69). Following the incubation, the cells were washed thrice and grown in culture media in the presence of 10 nM calcitriol or vehicle control.

### Real-time PCR assays

RNA was extracted 24 h after addition of calcitriol using RNeasy Mini Kit (Qiagen) as per manufacturer's protocol. DNsae-I treated (New England Biolabs) RNA (1 μg) was used for complementary DNA synthesis using iScript cDNA synthesis kit (Bio-Rad). Faststart essential DNA green master (Roche) was used for real-time PCR, with appropriate primers (Table S2). A standard curve was prepared to determine the absolute quantity of GAPDH, pcRNA, and HBV core promoter transcripts. The amount of pgRNA was calculated by subtractive analysis (pgRNA = core promoter transcripts − pcRNA). pgRNA and pcRNA levels were normalized to that of GAPDH (70, 71, 72).

### Luciferase assay

Cells seeded in 24-well plates were transfected with 0.49 μg of luciferase reporter construct (PGL3 vector carrying promoter of interest) and 0.01 μg of pRL-TK (internal control) per well. Vehicle or 10 nM calcitriol was added immediately after transfection. The cells were processed, and luminescence was measured 24 h after ligand treatment using the dual luciferase reporter assay system (Promega) according to manufacturer's protocol.

### In silico VDRE identification and conservation analysis

Whole-genome consensus sequences for each HBV genotype (“A” [n = 874], “B” [n = 1754], “C” [n = 1438], “D” [n = 1068], “E” [n = 302], “F” [n = 257], “G” [n = 39], and “H” [n = 25]) were generated using sequences available in the HBV database (38). This was done using MAFFT offline program with G-INS-i strategy and an in-house Python code (73, 74). The consensus core promoter of each genotype was screened for VDREs using another Python code developed in our laboratory (available on GitHub at: https://github.com/divyachoudhary2809/VDRE_). The code was designed to screen for DR-3 type VDREs with the consensus sequence RGDKYR (R = G or A, D = A, G, or T, K = G or T, and Y = C or T) tolerating up to one mismatch from this sequence, with the exception of G at the second position. This criterion was selected based on previous reports (75, 76) and our analysis of functionally verified VDREs. For conservation analysis, alignments for the VDRE-containing region in each genotype were generated using MAFFT and further analyzed using a python code (73, 74).

### Plasmid constructs and cloning

The greater-than-genome-length (1.3×) of the HBV genotype “D” cloned into pSLIRES-11 construct was a gift from Dr Syed Naqui Kazim (Jamia Millia Islamia, New Delhi, India). pSG5-hVDR and pSG5-hRXR used for *in vitro* translation in the gel shift assays were kind gifts from Dr Peter Jurutka (Arizona State University, USA) and Dr Christopher Sinal (Dalhousie University, Canada) (77, 78).

The regions corresponding to the (i) HBV core promoter, (ii) PreS1 promoter, (iii) PreS2/S promoter, and (iv) HBx promoter were amplified separately by PCR from the 1.3× HBV genome construct (genotype “D”) using appropriate primer pairs given in Table S2 (79). The promoter amplicons were digested and inserted between *KpnI* and *SacI* (New England Biolabs) sites upstream of the luciferase reporter gene in the pGL3-basic construct. The consensus HBV genotype “G” core promoter sequence (Fig. S5*A*) was purchased as a double-stranded synthetic fragment (GeneArt Strings; Thermo Fisher Scientific) and inserted between KpnI and XhoI (New England Biolabs) sites in the PGL3-basic construct for luciferase reporter assays.

Modification of the HBV core promoter in the PGL3-basic construct was done as described previously (80). Overlapping primers carrying the desired modifications so as to disrupt the VDRE cluster are provided in Table S2. A combination of a point mutation (G to A at 1740) and a deletion (T at 1739) introduced in the wildtype PGL3-core promoter disrupted the original sequences of all three VDREs in this cluster, while ensuring the overlapping Sp1-binding sites were intact (Fig. 3*A*).

### Gel-shift assay

Hybridized double-stranded oligonucleotides corresponding to the VDREs in (a) rat *Pit-1* gene promoter (a human gene known to contain VDREs and regulated by vitamin D; positive control) and (b) HBV-core VDRE cluster were procured from Integrated DNA Technologies. The oligonucleotides used were 5′ 6-FAM labeled or unlabeled (competitor oligonucleotide). The sequences of the probes used are as follows: rat Pit-1 VDRE—AAAACAGAAGTTCATGAGAGTTCATGGGGATT and HBV-core VDRE cluster—TAAAGACTGGGAGGAGTTGGGGGAGGAGATT. pSG5-hRXR construct, pSG5-hVDR construct, and the pSG5 vector were used to prepare hRXR alpha, hVDR, and cell extract (negative control) by *in vitro* translation using the TnT Quick Coupled Transcription/Translation System (Promega) as per the manufacturer's protocol. The IVT protein was incubated at 26 °C for 10 min in a binding buffer (10 mM Hepes [pH 7.9], 150 mM KCl, 1 mM dithiothreitol, 0.2 μg/μl of poly[dI-C], 5% glycerol) in a total volume of 20 μl. This was followed by addition of the desired 6-FAM probe to the IVT product to a final concentration of 5 nM and further incubation at 24 ^°^C for 30 min. The probe–protein complex was resolved on a nondenaturing 8% (w/v) polyacrylamide gel in 0.5× TBE (Himedia Laboratories Pvt Ltd). Cold unlabeled competitor oligonucleotides were added at the same time as the 6-FAM probe, when required as indicated.

### ChIP

HepG2 cells were grown in T75 flasks to a confluency of about 90% and transfected with 1.3× HBV genome construct. Vehicle or 10 nM calcitriol was added immediately after transfection. Cells were processed after 24 h using the EZ-ChIP Chromatin Immunoprecipitation Kit (Millipore) as per the manufacturer's protocol. Briefly, cells treated with 1% formaldehyde were washed, pelleted, and lysed to release chromatin. The cell lysate was sonicated using Bioruptor Plus Sonication System (Diagenode) for 4 × 10 cycles, for 30 s ON/30 s OFF at “high” setting to obtain DNA ranging from 200 bp to 1000 bp. The sheared chromatin was incubated with anti-VDR antibody (Abcam; ab3508), anti-RXRa antibody (Cell Signaling Technology; D6H10), or rabbit IgG (Thermo Fisher Scientific; 02-6102). Protein G Agarose beads were added to each sample the next day for 1 h at 4 °C to bind the antibody–chromatin complex, which was later eluted in the presence of 1% SDS and 0.1 M NaHCO_3_. Finally, the DNA–protein crosslinking was reversed in the presence of 200 nM NaCl at 65 °C for 4 h, and the DNA was subsequently purified using spin columns provided in the kit. Real-time PCRs were performed with primers listed in Table S2 to quantitate the immunoprecipitated target DNA.

### Virion estimation

HBV virion estimation was done using a technique previously developed in our laboratory, with a few modifications (81). HepG2, Huh7 (transfected with 1.3× HBV genome construct), HepG2.2.15, and HepG2-NTCP (infected with HBV) cells were grown in 6-well plates in the presence of 10 nM calcitriol or vehicle. The supernatant from each sample was collected 72 h after transfection and added to four wells of HBsAg ELISA plates (MONOLISA; Bio-Rad). About 150 μl of the supernatant was added to each well of the HBsAg ELISA plate, along with DNaseI (New England Biolabs) and incubated for 2 h at 37 °C for immunocapture of the viral particles. Each well was washed five times with 1× PBS (Himedia Laboratories Pvt Ltd) and further treated with 25 μl proteinase K per well. The captured virions were lysed, and the virion-associated DNA was isolated using QIAamp DNA Mini Kit (Qiagen) as per the manufacturer's suggested protocol. Virion-associated DNA was quantitated by RT-PCR using the primers given in Table S2.

### Estimation of secretory HBsAg and HBeAg

HepG2 and Huh7 cell lines were transfected with 1.3× HBV genome construct, whereas HepG2.2.15 contains stably integrated HBV. HepG2-NTCP cells were infected with HBV as described previously. Vehicle or 10 nM calcitriol was added immediately after transfection or infection, and the supernatant was collected after 48 h. The supernatant was appropriately diluted in 1× PBS to ensure absorbance values are in the linear range. Secreted HBsAg was quantitated using HBsAg ELISA plates (MONOLISA; Bio-Rad), whereas HBeAg was quantitated on HBeAg ELISA plates (Diasorin) as per the manufacturer's instructions (82).

### Statistical analysis

All data were generated with at least three independent experiments (n = 3). The data were analyzed using the Student's *t* test, and *p* values < 0.05 were considered significant.

## Data availability

The python code used for detection of VDREs is freely available for download at GitHub: https://github.com/divyachoudhary2809/VDRE_. All data are available in the main text or in the supporting information details. All constructs prepared in this article are available on request to the corresponding author.

## Supporting information

This article contains supporting information.

## Conflict of interest

The authors declare that they have no conflicts of interest with the contents of this article.
