# Supplementary material for: Vitamin D signaling inhibits HBV activity by directly targeting the HBV core promoter
Source: J Biol Chem. 2021 Sep 23;297(4):101233. doi: 10.1016/j.jbc.2021.101233 (PMC8517215; doi:10.1016/j.jbc.2021.101233)
Supplement: Figures S1–S5 and Tables S1 and S2 [file mmc1.pdf]

# SUPPORTING INFORMATION

## Vitamin D signaling inhibits Hepatitis B Virus (HBV) activity by directly targeting the HBV Core promoter

Shivaksh Ahluwalia[1], Divya Choudhary[2], Purnima Tyagi[3], Vijay Kumar[3] and Perumal Vivekanandan[1,*]

[1]Kusuma School of Biological Sciences, Indian Institute of Technology Delhi, New Delhi, India

[2]Department of Chemical Engineering, Indian Institute of Technology Delhi, New Delhi, India

[3]Department of Molecular and Cellular Medicine, Institute of Liver and Biliary sciences, New Delhi, India

*Address correspondence to Perumal Vivekanandan at vperumal@bioschool.iitd.ac.in

Present Address for Divya Choudhary: Department of Biochemistry, University of Oxford, South Parks Road, Oxford, OX1 3QU, United Kingdom.

## LIST OF SUPPORTING INFORMATION

**Fig. S1.** HBV Promoters and transcriptome map

**Fig. S2.** Vitamin D signaling-pathway is active in hepatic cell lines

**Fig. S3.** Calcitriol does not regulate the HBV genotype 'G' core promoter, which lacks VDREs

**Fig. S4.** Gel shift assay using a fragment of Rat-Pit 1 promoter as a procedural positive control

**Fig. S5.** HBV-Core promoter sequences

**Table S1.** : The sequence and position of VDREs present in HBV genotype 'D' core promoter used in this study

**Table S2.** Primer sequences used in this study

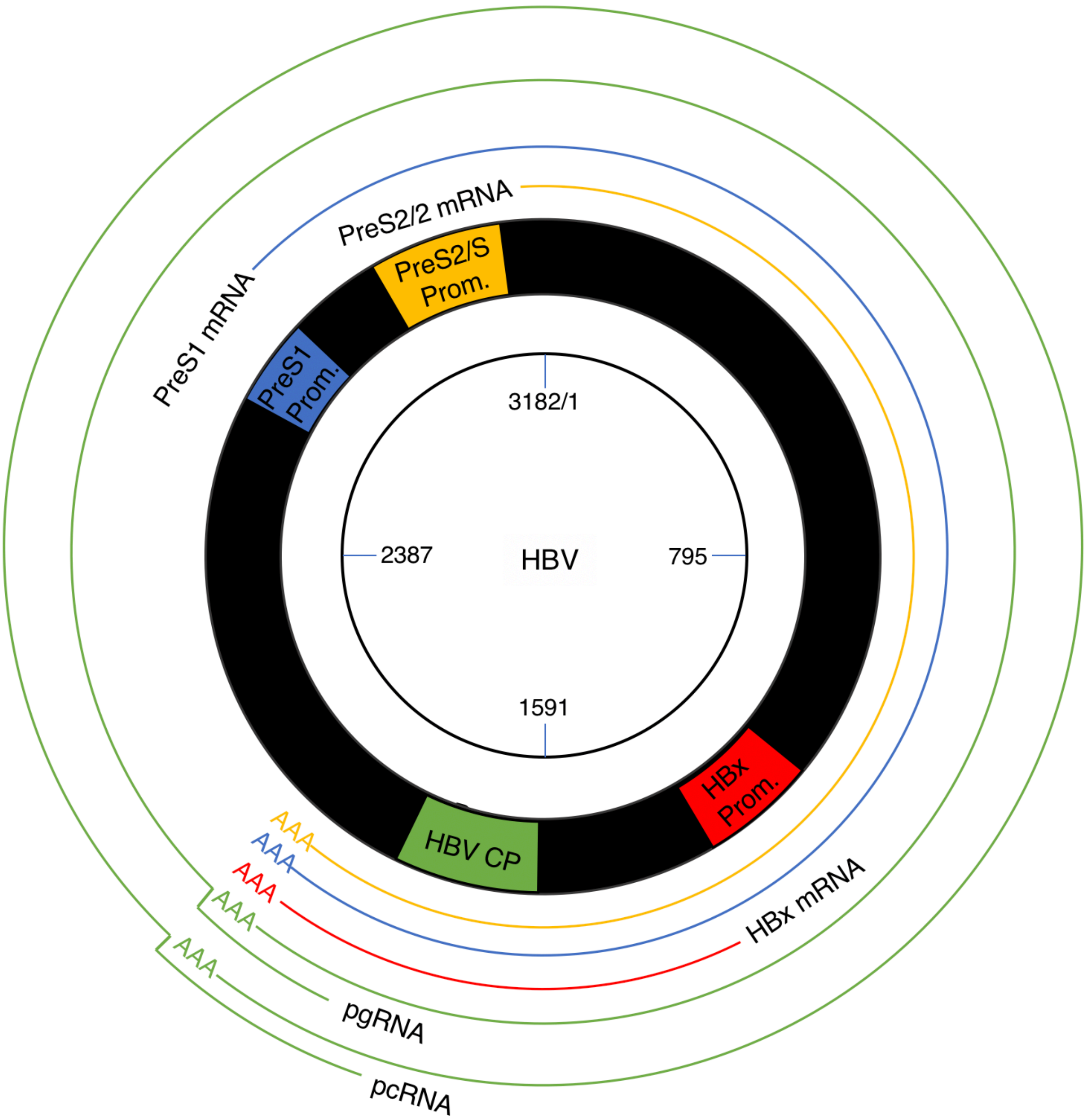


**Fig. S1. HBV Promoters and transcriptome map.** The above illustration depicts the genome of HBV Genotype ‘D’ (Black), along with the conventional numerical coordinates in which the unique EcoRI site is positioned at 3182/1 (inner-most circle). The relative position of the 4 HBV promoters and their canonical transcripts have been shown on the HBV genome. The HBx promoter (red box) transcribes the 0.7 kb HBx mRNA (red line). The preS1 promoter (blue Box) and PreS2/S promoter (yellow box) give rise to the 2.4 kb PreS1 mRNA (blue line) and 2.1 kb PreS2/S mRNA (yellow line) respectively, which encode HBV surface proteins. The core promoter (green box) controls the transcription of the 3.5 kb HBV pgRNA and HBV pcRNA (green lines).

The TSS for pgRNA lies downstream to that of the pcRNA, and hence the pgRNA is marginally shorter than the pcRNA. All the transcripts terminate with a common 3'-polyadenylation signal downstream of the core promoter (AAA).

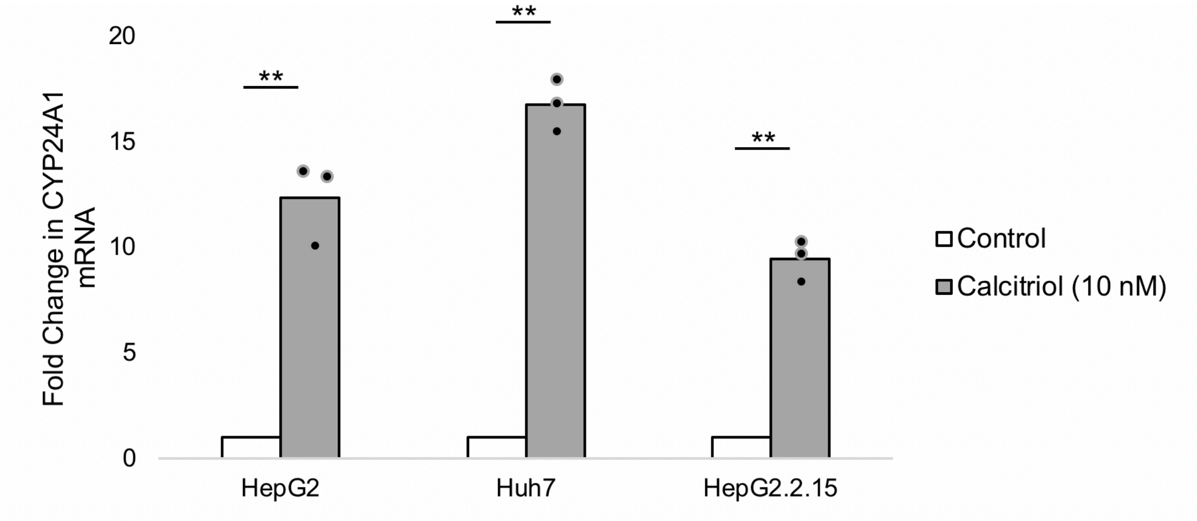


**Fig. S2. Vitamin D signaling-pathway is active in hepatic cell lines.** CYP24A1 mRNA was measured by qPCR after 24 hour treatment with 10nM calcitriol or vehicle-control (without calcitriol) in hepatic cell lines, HepG2, Huh7 and HepG2.2.15. The induction of CYP24A1 mRNA on ligand addition demonstrates that the vitamin D signaling pathway is active in all the three cell lines. All data are means ±S.D. for three independent experiments (n=3). *$P$<0.05, **$P$<0.01, *NS*: not significant were analysed by paired Student's *t*-test.

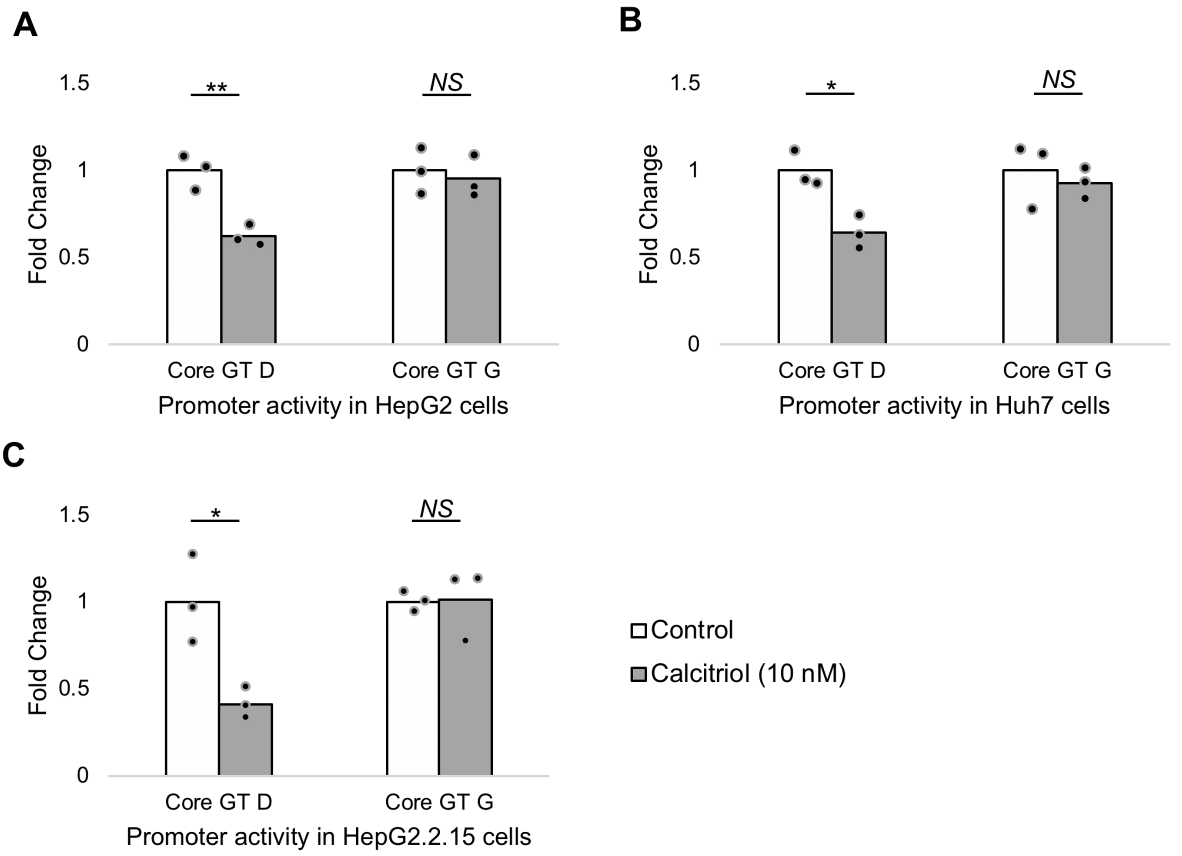


**Fig. S3. Calcitriol does not regulate the HBV genotype 'G' core promoter, which lacks VDREs.** The genotype 'G' and genotype 'D' core promoters were cloned separately, upstream of the luciferase reporter gene in the PGL3-basic construct (see methods section for details). The activity of the promoters were tested in the presence of vehicle-control (without calcitriol) or 10nM calcitriol in (**A**) HepG2, (**B**) Huh7 and (**C**) HepG2.2.15 by luciferase assays. The activity of the HBV genotype 'D' core promoter (Core GT D), carrying the identified VDRE-cluster, was significantly reduced in the presence of calcitriol; however, the activity of HBV genotype 'G' core promoter lacking VDREs was not affected by addition of the ligand. This highlights the importance of the identified HBV VDRE-cluster in calcitriol-mediated inhibition of the HBV-core promoter. All data are means ±S.D. for three independent experiments (n=3). **P*<0.05, ***P*<0.01, *NS*: not significant were analysed by paired Student's *t*-test.

**A**

5′ 3′

*6-FAM*-aaaacagaAGTTCAtgagAGTTCAtggggatt

**B**

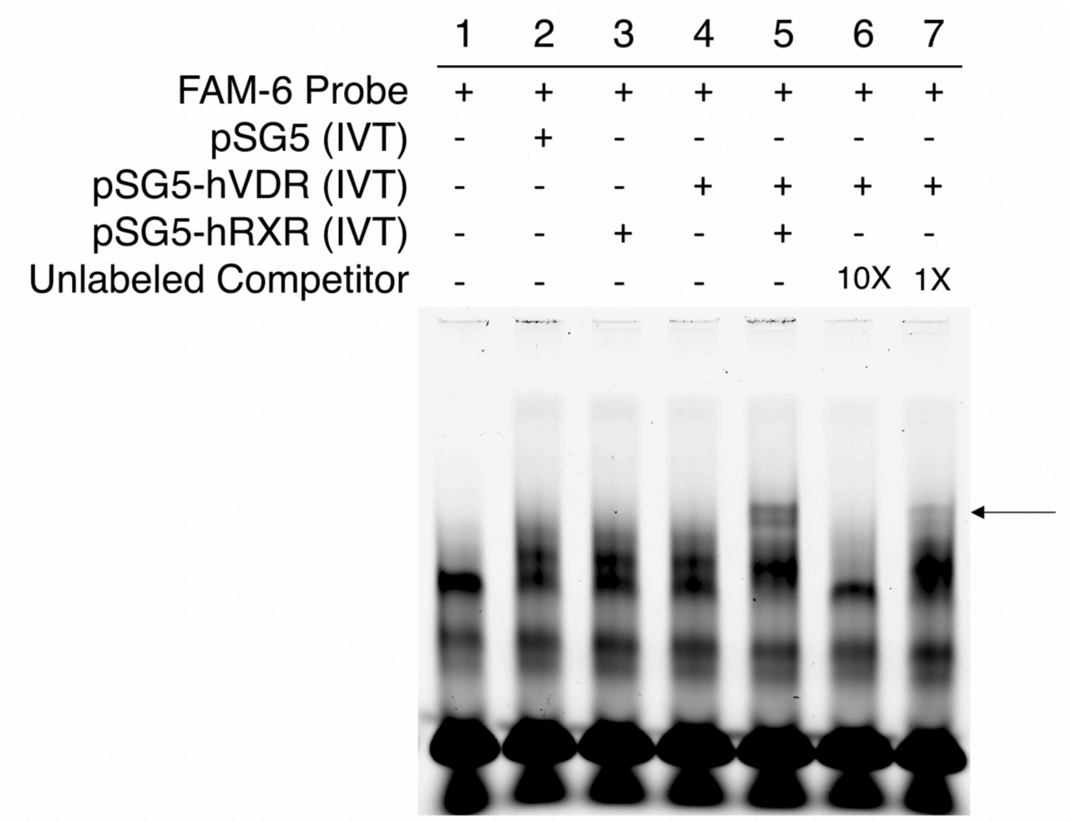


**Fig. S4. Gel shift assay using a fragment of Rat-Pit 1 promoter as a procedural positive control.** (**A**) A fragment of the Rat-Pit 1 promoter having an established DR4-type VDRE (Underlined). The two hexameric core sequences (capitalized) serve as the RXR and VDR binding sites. The probe was labelled with FAM-6 fluorescent dye at the 5’ end and used a procedural positive control for the gel shift assay. (**B**) *In vitro* translated VDR and RXR were incubated with the probe and unlabeled competitor as indicated. The complex was then resolved on a native polyacrylamide gel as per the protocol described in the methods section. VDR and RXR together bind to the probe as

suggested by the indicated band (*see arrow)* in lane 5. Unlabeled competitor of the same sequence as the probe was added as indicated. It competes with the probe to bind VDR-RXR heterodimer and reduces the visible complex formed in a dose dependent manner, demonstrating the specificity of the binding reaction.

**A**

**>HBV Genotype D Core Promoter**
CCAAATATTGCCCAAGGTCTTACATAAGAGGACTCTTGGACTCTCAGCAATG
TCAACGACCGACCTTGAGGCATACTTCAAAGACTGTTTGTTTAAAGACTGG
GAGGAGTTGGGGGAGGAGATTAGGTTAAAGGTCTTTGTACTAGGAGGCTGT
AGGCATAAATTGGTCTGCGCACCAGCACCATGCAACTTTTTCACCTCTGCCT
AATCATCTCTTGTT

**B**

**>HBV Genotype G Core Promoter**
TCATCATCTGCCAAGGCAGTTATATAAGAGGACTCTTGGACTGTTTGTTATGT
CAACAACCGGGGTGGAGAAATACTTCAAGGACTGTGTTTTTGCTGAGTGGG
AAGAATTAGGCAATGAGTCCAGGTTAATGACCTTTGTATTAGGAGGCTGTAG
GCATAAATTGGTCTGCGCACCAGCACCATGTAACTTTTTCACCTCTGCCTAA
TCATCTCTTGTT

**Fig. S5. HBV-Core promoter sequences cloned in PGL3-basic.** Sequence of HBV-core promoter (**A**) genotype 'D' and (**B**) genotype 'G'. These sequences were cloned into the PGL3-basic construct to study their activity in the presence of calcitriol as detailed in the methods section

| S. No. | VDRE Sequence | Start | Stop |
|---|---|---|---|
| 1 | AGACTGGGAGGAGTT | 1725 | 1739 |
| 2 | GGAGGAGTTGGGGGA | 1731 | 1745 |
| 3 | GGAGTTGGGGGAGGA | 1734 | 1748 |

**Table S1. : VDREs in HBV genotype 'D' core promoter.** The sequence and position (numerical coordinates on HBV genome as per GenBank Sequence V01460.1) of the DR3-type VDREs clustered in HBV genotype 'D' core promoter used in this study.

| Name | Forward or Reverse Primer | Sequence |
|---|---|---|
| CYP24A1 qPCR | FP | TGGAAGGCCTATCGCGACTA |
| | RP | GGACCCGCTGCCAGTCTT |
| GAPDH qPCR | FP | TGCACCACCAACTGCTTAGC |
| | RP | GGCATGGACTGTGGTCATGAG |
| HBV Core Promoter Cloning | FP | ATGGTACCCCAAATCTTGCCCAAGATCT |
| | RP | ATGAGCTCAACAAGAGATGATTAGGCAG |
| HBV HBx Promoter Cloning | FP | ATGGTACCAAGTGTTTGCTGACGCAA |
| | RP | ATGAGCTCCATGGAAACGATGTATATTT |
| HBV PreS1 Promoter Cloning | FP | ATGGTACCACATCTAGTTAATCATTACT |
| | RP | ATGAGCTCCTATGTGTTGTTTCTCT |
| HBV preS2/S promoter Cloning | FP | ATGGTACCGATTGGGACCTCAACCCACA |
| | RP | ATGAGCTCGATCTTGAAGAGTTTGGT |
| HBV Core Mutant | FP | TTCGTCTCGGGGGAGGAGATTAGATTAAAGG |
| | RP | TTCGTCTCTCCCCTACTCCTCCCAGTCTT |
| Core promoter transcripts qPCR | FP | CACCTCTGCCTAATCATC |
| | RP | GGAAAGAAGTCAGAAGGCAA |
| pcRNA qPCR | FP | GGTCTGCGCACCAGCACC |
| | RP | GGAAAGAAGTCAGAAGGCAA |
| HBV-core promoter (ChIP) | FP | CCACTTCTTGCCCAAGGTC |
| | RP | GATTAGGCAGAGGTGAAAAAGTTGC |
| HBV PreS1 Promoter (ChIP) | FP | GGGTATTAAACCTTATTATCCAGAAC |
| | RP | CCCACAAAATGAGGCGCTA |
| Cyp24A1 Promoter (ChIP) | FP | GTCCAGGCTGGGGGTATCTG |
| | RP | CGCAGAGGAGGGCGGAGTGG |

| Name | Forward or Reverse Primer | Sequence |
|---|---|---|
| Virion qPCR | FP | GGTCTGCGCACCAGCACC |
| | RP | GAACTTTAGGCCCATATTAGTG |
| HBV DNA | FP | CTTCATCCTGCTGCTATGCCT |
| | RP | AAAGCCCAGGATGATGGGAT |

**Table S2. : Primer sequences used in this study.** For details please refer to the methods section.
